# Embryonic exposure to corticosterone modifies the juvenile stress response, oxidative stress and telomere length

**DOI:** 10.1098/rspb.2011.1913

**Published:** 2011-11-09

**Authors:** Mark F. Haussmann, Andrew S. Longenecker, Nicole M. Marchetto, Steven A. Juliano, Rachel M. Bowden

**Affiliations:** 1 Department of Biology, Bucknell University, Lewisburg, PA, USA; 2 School of Biological Sciences, Illinos State University, Normal, IL 61790-4120, USA

**Keywords:** stress, maternal effects, bird, corticosterone, oxidative stress, telomere

## Abstract

Early embryonic exposure to maternal glucocorticoids can broadly impact physiology and behaviour across phylogenetically diverse taxa. The transfer of maternal glucocorticoids to offspring may be an inevitable cost associated with poor environmental conditions, or serve as a maternal effect that alters offspring phenotype in preparation for a stressful environment. Regardless, maternal glucocorticoids are likely to have both costs and benefits that are paid and collected over different developmental time periods. We manipulated yolk corticosterone (cort) in domestic chickens (*Gallus domesticus*) to examine the potential impacts of embryonic exposure to maternal stress on the juvenile stress response and cellular ageing. Here, we report that juveniles exposed to experimentally increased cort *in ovo* had a protracted decline in cort during the recovery phase of the stress response. All birds, regardless of treatment group, shifted to oxidative stress during an acute stress response. In addition, embryonic exposure to cort resulted in higher levels of reactive oxygen metabolites and an over-representation of short telomeres compared with the control birds. In many species, individuals with higher levels of oxidative stress and shorter telomeres have the poorest survival prospects. Given this, long-term costs of glucocorticoid-induced phenotypes may include accelerated ageing and increased mortality.

## 1. Introduction

Vertebrates respond to stress by activating a suite of integrated physiological response mechanisms termed a stress response, of which a key component is the hypothalamic-pituitary-adrenal (HPA) axis. Activation of the HPA axis enhances survival by releasing glucocorticoid hormones that act to mobilize energy stores, inhibit unnecessary physiological functions and regulate behaviours that control energy intake and expenditure [1,2]. Once homeostasis has been restored, glucocorticoids quickly return to baseline levels. However, exposure to repeated or prolonged stressors gives rise to chronic stress. This dysregulation of the HPA axis often results in increased exposure to glucocorticoids through an elevation in baseline levels, heightened acute responses and a protracted decline to baseline levels during stress recovery ([3], but see [4]). The resultant overexposure to glucocorticoid hormones has been shown to suppress somatic growth, immune function, reproduction and, ultimately reduce survival [1–3].

Stress experienced by mothers can expose foetuses to maternally derived glucocorticoids through the placenta in mammals [5] and through their presence in eggs of oviparous species [6]. Across diverse taxa, this prenatal stress can broadly impact physiology and behaviour [7,8]. The transfer of maternal glucocorticoids to offspring may be an inevitable cost associated with poor environmental conditions. Indeed, prenatal stress often has effects reminiscent of those seen in chronic stress, including a reduction in hatch weight, reduced growth and compromised immunity [9–11]. However, maternal glucocorticoids may benefit offspring by serving as a maternally mediated cue that alters offspring phenotype in preparation for a stressful environment [8,12]. For example, in both birds [10,12,13] and mammals [7] maternally derived glucocorticoids can alter the responsiveness of the HPA axis, thereby influencing how offspring respond to stressful situations later in life. Early embryonic exposure to glucocorticoids is likely to have both costs and benefits that are paid and collected over different developmental time periods, and whether these are adaptive or merely a physiological constraint is probably based on a species' particular life history [8]. Although most studies have focused on the short-term effects of maternal glucocorticoids, long-term effects are beginning to be explored [14].

A recent link between elevated glucocorticoids and accelerated cellular ageing in humans suggests that increased embryonic exposure to maternal glucocorticoids may have long-term survival costs. Both chronic stress [15] and elevated glucocorticoids [16] are associated with increased oxidative stress and short telomeres. The use of oxygen for efficient energy metabolism can produce reactive oxygen species (ROS), which in turn can result in oxidative stress. While limited amounts of ROS serve important functions as regulators in signal processing, at higher concentrations, ROS result in oxidative damage. Organisms have evolved mechanisms, such as antioxidant defences, to stop the propagation of ROS and the oxidative damage that they create [17]. Telomeres, the evolutionarily conserved caps found at the ends of chromosomes that protect genomic integrity [18], are particularly vulnerable to ROS attack, which can result in their shortening (reviewed in [19]). For this reason, the rate of telomere loss may be a useful biomarker of chronic oxidative stress [20]. Although telomeres can be restored through the enzyme telomerase, if shortened to a critical length, the shortened telomeres induce a permanent arrest in the cell cycle through a process called cellular senescence [18]. Accumulating evidence suggests that reduction in telomere length is a component of the ageing phenotype as well as a risk factor in a large number of diseases [18]. Telomere length appears to predict remaining lifespan in humans ([21–23], but see [24]), and in wild birds, individuals with the shortest telomeres or the highest loss rate have the poorest survival prospects [25–28].

Searching for links between stress hormones and ageing and how they impact organismal fitness requires integrative work across the fields of endocrinology, gerontolgy and evolutionary biology. Given the growing evidence that stress hormones can influence oxidative stress [29], and that oxidative stress is associated with reduced survival [30], it is possible that maternally derived glucocorticoids shape the ageing trajectory of offspring. In this study, we manipulated yolk corticosterone (cort) in domestic chickens (*Gallus domesticus*) to better understand the relationships between yolk glucocorticoids and cellular ageing processes. We had two hypotheses:

— exposure to exogenous cort during embryonic development alters the functioning of the HPA axis during an acute stress response post-hatch, and
— exposure to exogenous cort during embryonic development influences cellular ageing processes.

The first hypothesis is based on the knowledge that elevated maternal glucocorticoids can permanently modify the development and subsequent function of the HPA axis in offspring. The direction of this modification is quite variable and depends not only on the timing, duration and magnitude of glucocorticoid exposure, but also varies by species and sex [7]. These processes are probably best understood in the rat model, where maternal cort in developing rats reduce the number of glucocorticoid type I and II receptors in the hippocampus [31]. This reduction results in impaired negative feedback control of cort secretion, which often produces higher baseline levels and a prolonged duration of the stress response [7]. Given this, we predicted that, compared with controls, chicks from cort-injected eggs would have higher baseline cort levels, higher total exposure to cort during an acute stress response, and a slower cort decline during stress recovery (i.e. a higher cort concentration at the last sampling time).

Our second hypothesis follows the recent work highlighting a link between glucocorticoids and cellular ageing. Elevated glucocorticoids may impact cellular ageing through a number of diverse mechanisms, including an increase in the generation of ROS, disabling antioxidant defences, or accelerating telomere loss [19,32]. Elevated glucocorticoids in chickens [33,34], kestrels [35] and owls [36] increase oxidative damage, while chronic administration of glucocorticoids in rats causes increased lipid peroxidation and decreased antioxidant activity [37,38]. We predicted that, compared with controls, chicks from cort-injected eggs would have higher baseline levels of oxidative stress, characterized by greater oxidative damage and deficient antioxidant capacity. Because high levels of oxidative stress can hasten telomere shortening, we also predicted that cort-injected birds would have a higher proportion of short telomeres compared with controls.

## 2. Methods

### (a) Study species

Eighty fresh Bovans White chicken eggs were obtained from Centurion Poultry, Inc., (Milton, PA, USA) in March 2010. The eggs were collected from hens that were approximately 60 weeks old. Eggs were stored at 16–18°C until injected as described below. All procedures were conducted with approval from the Bucknell University Institutional Animal Care and Use Committee.

### (b) Injection, incubation and hatching

The 80 eggs were weighed and, to minimize differences in initial egg size among our treatment groups, the 10 lightest and 10 heaviest eggs were removed from the experiment. The remaining 60 eggs were assigned to control, low- and high-cort groups (*n* = 20 each), with the same means ± s.d. for egg mass. In each of these groups, five eggs were used for yolk cort analyses and were not incubated, leaving 15 eggs per treatment. Given that previous experiments report hatch rates between 18 and 81 per cent following yolk injection ([11], reviewed in [39]), this sample size was chosen to provide a minimum of five birds per treatment. In our experiment, hatch rates for each group were 47 per cent (7 of 15 injected eggs hatched in each treatment).

The embryo's adrenal cortex is able to secrete cort in response to adrenocorticotropic hormone starting on embryonic day 5, but prior to then embryos may be exposed to maternal steroids in the yolk. In chickens, endogenous yolk cort levels are highly variable ranging from 0.8 to 27.3 ng cort g^−1^ yolk [40,41]. We chose two experimental concentrations that would elevate yolk cort while attempting to remain within physiological levels: low cort (0.15 µg per 50 µl vehicle or 5 ng g^−1^ yolk) and high cort (0.3 µg per 50 µl vehicle or 10 ng g^−1^ yolk). Cort injection successfully elevated yolk levels in the low- and high-cort groups relative to controls. Average yolk cort level for control eggs was 2.13 ± 1.21 ng g^−1^, for low-cort eggs was 5.13 ± 0.76 ng g^−1^, and for high-cort eggs was 10.21 ± 0.90 ng g^−1^.

Injection order was chosen randomly and a small hole was drilled through the shell in the middle of the long axis and about one-third down from the top surface of the egg. On embryonic day 0, all eggs were injected into the yolk by advancing a Hamilton syringe into the hole until the yolk membrane was penetrated (approx. 20 mm). This technique has been previously verified in our hands. Eggs in the control group were injected with the sesame oil vehicle (50 µl) and eggs in the cort groups were injected with cort in sesame oil. Eggs sat at room temperature for 1 h after injection and then five eggs from each group were placed at −20°C for subsequent yolk cort analysis. The remaining 45 eggs were placed in a GQF 1502 Digital Sportsman incubator (GQF Manufacturing Co., GA, USA) and maintained at 37.5°C and 65 per cent humidity while being turned every 2 h. Three days before hatching, eggs were transferred to a GQF 1550 Digital Hatcher at 37°C and 80 per cent humidity.

After hatching, chicks were marked with numbered wing bands and placed into mixed treatment cohorts of seven chicks, each with at least two chicks from each treatment in each cohort. All cohorts were reared in a single room in Brower brooders that were maintained at 35°C for the first week and 32°C thereafter. Birds received ad libitum food (Start & Grow Sunfresh Recipe, Purina Mills, MO, USA) and water. Mass, wing length, and tarsus length measurements were taken every day within the same hour. Sex was initially determined by morphological characters including wing length and comparison of primary and secondary feathers. Sex was later confirmed by the presence of a secondary sex character (i.e. comb size) and macroscopic inspection of the gonads.

At 25 days of age, we measured activity of the HPA axis by obtaining a stress series. Approximately 75 µl of blood was taken from the alar vein within 3 min (termed baseline), and again at 15 and 40 min from initial handling of the chicks. Between blood draws chickens were restrained in breathable, cotton bags with minimal stimulation. Blood samples were kept on ice until completion of the stress series and thereafter blood was spun down at 3000*g* for 10 min. Plasma was removed and frozen in aliquots at −20°C. The red blood cells were resuspended in cryoprotectant buffer (10% dimethyl sulphoxide, 90% newborn bovine serum) and frozen at −20°C. Samples from less than 3, 15 and 40 min were used for cort assays while only samples from less than 3 and 40 min were used for oxidative stress analyses.

### (c) Yolk and plasma cort assay

Frozen eggs were slowly thawed at room temperature until they could be cut and separated into shell, albumen and yolk. Homogenized yolk samples and plasma, collected as described above, were stored at −20°C until radioimmunoassay (RIA).

Cort levels were determined by RIA [42]. Samples were prepared by diluting 48.8–50.9 µg of yolk in 500 µl of water, or 16–50 µl of plasma with water to a total volume of 400 µl. Tritiated tracer (2000 cpm; NET 399, Perkin Elmer, Boston, MA, USA) was added to each sample for calculating recovery values. Yolk samples were extracted with 6 ml of 30 : 70 petroleum ether : diethyl ether and subjected to celite chromatography. Cort was eluted using 50 per cent ethyl acetate : isooctane, and samples were resuspended in 550 µl phosphate buffered saline with gelatin (PBS-gelatin). Plasma was extracted with 4 ml diethyl ether and resuspended in 550 µl PBS-gelatin, but was not run through celite columns [43]. Yolk and plasma samples were run in separate competitive-binding RIAs using a specific antibody for cort (20-CR45; Fitzgerald Industries, Acton, MA, USA). Steroid concentrations were calculated and compared with a standard curve that ranged from 7.81 to 2000 pg for yolk and 1.96 to 2000 pg for plasma. Recovery values averaged 58 per cent for yolk and 79 per cent for plasma. Intra-assay coefficient of variation (CV) was 3.5 per cent for yolk. For plasma, intra-assay CVs were 2.8 and 4.7 per cent, and inter-assay CV was 7.8 per cent.

### (d) Oxidative stress and telomere length

We assessed reactive oxygen metabolites (ROMs) and total plasma antioxidant capacity (TAC) from the less than 3 min (PRE) and the 40 min (POST) stress series blood samples. ROMs were measured using the d-ROMs test, which measures the level of hydroperoxides, compounds that signal lipid and protein oxidative damage (Diacron International, Grosseto, Italy). Levels of ROMs in the plasma change quickly as both a consequence of the release of free radicals and because ROMs are metabolized rapidly [30]. We diluted 10 µl of plasma in 200 µl of the provided acidic buffer solution, followed by incubation for 75 min at 37°C. Absorbance was measured at 490 nm (BioTek ELx800, VT, USA) and we calculated ROMs concentrations (in millimolar of H_2_O_2_ equivalents) from these absorbencies. All samples were run in a single assay and duplicate measurements of samples within the assay showed an intra-assay CV of 2.91 per cent.

TAC was measured using the OXY-adsorbent test, which measures the effectiveness of the blood antioxidant barrier by quantifying its ability to cope with oxidant action of hypochlorous acid (HClO; Diacron International, Grosseto, Italy). We diluted 10 µl plasma in 990 µl of distilled water; we then mixed 5 µl of this diluted plasma with 195 µl of the provided HClO solution and continued by following the manufacturer's instructions. We measured absorbance at 490 nm (BioTek ELx800) and we calculated TAC (in millimolar of HClO neutralized). All samples were run in a single assay and duplicate measurements of samples within the assay showed an intra-assay CV of 2.57 per cent. For more details regarding the ROMs and TAC assays, see Costantini *et al*. [44].

Telomeres were measured with the telomere restriction fragment (TRF) assay and the procedure was carried out according to previous studies [45]. Briefly, DNA was extracted in 0.8 per cent agarose plugs, digested with proteinase K, followed by restriction digestion with 15 U of *Hinf*I, 75 U of *Hae*III and 40 U of *Rsa*I at 37°C. Plugs were loaded into a 0.8 per cent non-denaturing agarose gel. DNA was separated using pulsed field gel electrophoresis (3 V cm^−1^, 0.5–7.0 s switch times, 14°C) for 21 h, followed by in-gel hybridization at 37°C overnight with a radioactive-labelled telomere-specific oligo (CCCTAA)_4_. Hybridized gels were placed on a phosphorscreen (Amersham Biosciences, Buckinghamshire, UK), which was scanned on a Storm 540 Variable Mode Imager (Amersham Biosciences). We used densitometry (ImageQuant 5.03v and ImageJ 1.42q) to determine the position and the strength of the radioactive signal in each of the lanes compared with the molecular marker (1 kb DNA Extension Ladder; Invitrogen, CA, USA).

A major advantage of the TRF method is that it provides information on the entire TRF distribution. Frequency distributions of telomere length for each individual were produced following Kimura *et al*. [23]. The resulting plots allow visualization of the relative abundances of TRFs at each molecular weight (MW) providing useful information on where differences in telomere length occur among groups. For each individual bird, area under the optical density curve was calculated in 1 kb intervals from 1–40 kb (the limits of our molecular marker), and each interval was divided by the area under the curve for the entire distribution. The background was fixed as the nadir of the low MW region on the gel (less than 1 kb). Relative abundances of TRFs in each of the MW classes were log transformed and then fit by least-squares fourth-order polynomial regression with the MW classes [1–40].

### (e) Statistics

For growth, plasma cort and oxidative stress analyses, we tested the effects of sex, treatment and their interaction on our response variables. Preliminary analyses also included effects of cohort and all its interactions; none of these effects was significant and was excluded from final analyses. Exponential growth rates for mass, tarsus length and wing length between days 4 and 21 were estimated as:
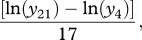
where *y*_*i*_ are the values of a given variable for day *i*, and 17 is the number of days. We used ANOVAs to compare these growth rates for each variable for the three treatments. Change in plasma cort levels over the acute stress response was analysed using repeated-measures multivariate analysis of variance (MANOVA) with time as the repeated measure. Data were square-root transformed for analysis, and we report *F*-statistics derived from Pillai's Trace.

Effects of cort on ROMs and TAC were tested in three separate MANOVAs (less than 3 min, 40 min and the change between 3 and 40 min within an individual; *F*-statistics from Pillai's Trace), each with two dependent variables (ROMs and TAC). Change was quantified as: abundance at 40 min—abundance at 3 min. In addition, to determine if ROMs or TAC changed over the course of the stress response regardless of treatment group, we also tested whether there was a change in ROMs or TAC over time for all observations in all groups (ignoring treatment, sex and their interaction).

We assessed the effects of cort treatment on the distribution of TRFs using MANOVA with TRF relative abundances as dependent variables and treatment (control, low cort and high cort) as the independent variable. Our sample size of seven individuals per treatment limited degrees of freedom and made it necessary to limit analysis to 12 of the 40 TRF intervals as dependent variables (1–5 kb, and then every 5 kb thereafter). These intervals were chosen to enable us to compare treatments across the entire TRF distributions, while also providing more information at the low MW regions where short telomeres lead to telomere dysfunction. We analysed data using parametric MANOVA, and also using randomization MANOVA with 9000 randomizations [46], to determine if statistical results were robust to any departures from assumptions of parametric MANOVA. Because the randomization procedure uses Wilks' Lambda, we used this same statistic for the parametric MANOVA. For MANOVAs, we used standardized canonical coefficients (SCCs) to interpret significant effects [47].

All statistical analyses were conducted in SAS v. 9.2 (SAS Institute, Cary, NC, USA) except for the randomization MANOVA, which was done using RT v. 2.0 (West, Inc.). We used *α* = 0.05 for all tests of main effects and interactions. Any significant effects (sex, treatment, time and any interactions) were further tested using pairwise contrasts with a Bonferroni correction for multiple tests, resulting in a comparison wise *α* = 0.017 for pairwise contrasts. We report means ± standard error of the mean (s.e.m.).

## 3. Results

Our analysis of growth rates failed to detect any effects of sex, treatment or their interaction on mass (table 1). Neither treatment nor the treatment × sex interaction were significant for tarsus or wing length growth rate, but there were significant effects of sex on both variables (table 1), with tarsus and wing length growth for males greater than the same measures for females.

**Table 1.** ANOVA results for growth rates of mass, tarsus length and wing length in juvenile chickens. (Rates were estimated over a 17 day period (see text) for control, low-cort and high-cort birds (all groups *n* = 7). Significant effects are highlighted in bold.)

		mass		tarsus length		wing length	
source	d.f.	*F*	*p*	*F*	*p*	*F*	*p*
sex	1	1.02	0.328	**6.11**	**0.026**	**15.43**	**0.001**
treatment	2	1.91	0.183	2.65	0.104	0.43	0.657
sex × treatment	2	0.82	0.459	0.22	0.807	0.28	0.762
error	15						

**Figure 1.**
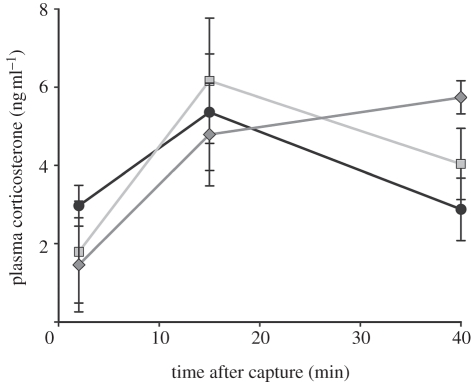
The effect of elevated embryonic corticosterone on plasma corticosterone concentration (mean ± s.e.m.) during an acute stress response in juvenile chickens (all groups, *n* = 7). Filled circles with solid line, control; squares with solid line, low cort; filled diamonds with solid line, high cort.

Repeated measures MANOVA yielded a significant treatment × time interaction for plasma cort levels (Pillai's Trace: *F*_4,30_ = 3.23, *p* = 0.026). While baseline cort did not differ among treatments, birds in all treatments responded by increasing cort levels between the baseline and 15 min samples. Levels in the high-cort group continued to increase across the stress response series, whereas levels in the control and low-cort groups were declining by 40 min (figure 1). Only the first eigenvector was significant (*p* = 0.028) in the MANOVA, accounting for 78 per cent of the variation in the time courses of plasma cort. The SCCs indicated that the change from 15 to 40 min contributed most to this significant treatment × time interaction (SCC = 1.179), but that there is also a substantial contribution from baseline to 15 min (SCC = 0.879). None of the multivariate pairwise contrasts for the treatment × time interaction was significant after correcting for multiple tests (electronic supplemental material, table S1), but graphical presentation of the data (figure 1) suggests a tendency for the high-cort group to respond differently than the other two groups. There were no statistically significant effects of sex, sex × treatment interaction or sex × time × treatment interaction on plasma cort levels for any sampling period.

**Figure 2.**
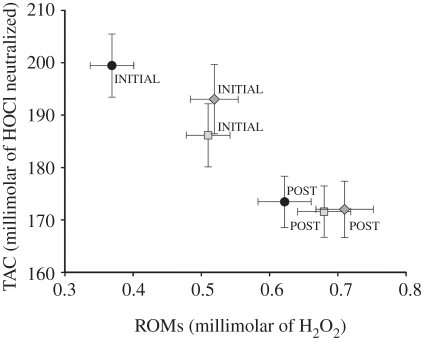
Relationships between reactive oxygen metabolites (ROMs) and total antioxidant capacity (TAC) within treatment groups at the initiation (INITIAL) and after (POST) an acute stressor. Least square mean ± s.e.m. plotted (all groups, *n* = 7). Filled circles, control; squares, low cort; diamonds, high cort.

Initial levels of both ROMs and TAC differed with cort treatment (Pillai's Trace: *F*_4,30_ = 3.61, *p* = 0.016; figure 2 and electronic supplementary material, table S2). Multivariate pairwise contrasts revealed that both cort-treated groups differed from the controls (*p* < 0.007), but not from one another (*p* = 0.75). This pattern was primarily attributable to an initially higher level of ROMs in the cort-treated chicks (ROMs SCC = 1.41, TAC SCC = −1.05, figure 2 and electronic supplementary material, table S2). The treatment groups did not differ in ROMs or TAC at 40 min post-initiation of the stress response (Pillai's Trace: *F*_4,30_ = 0.66, *p* = 0.62). Treatment groups also did not differ in the change over the stress response period (Pillai's Trace: *F*_4,30_ = 1.89, *p* = 0.14). However, across all groups, both ROMs and TAC changed significantly over the 40 min stress response period, but in opposite directions, with ROMs increasing and TAC decreasing (Pillai's Trace: *F*_2,14_ = 129.76, *p* < 0.0001; figure 2).

**Figure 3.**
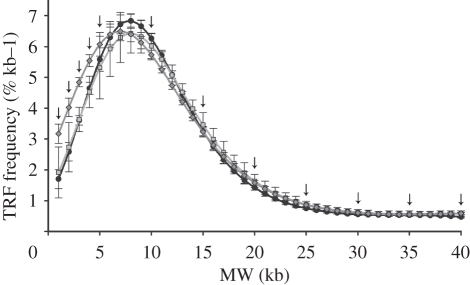
The effect of elevated embryonic corticosterone on telomere restriction fragment (TRF) length of juvenile chickens measured at 21 days of age. Plotted are mean (± s.e.m.) TRF frequency distributions from chicken erythrocytes against molecular weight (MW). The data are log transformed and then fit by least-squares fourth-order polynomial regression using the equation: log(freq) = *ß*_0_ + *ß*_1_ (MW) + *ß*_2_ (MW − medianMW)^2^ + *ß*_3_ (MW − medianMW)^3^ + *ß*_4_ (MW − medianMW)^4^. Analysis was performed on the 12 TRF intervals denoted by the arrows (see text; all groups *n* = 7). Filled circles with solid, control; squares with solid line, low cort; diamonds with solid line, high cort.

There were significant multivariate differences among the TRF frequency distributions for the treatment groups in parametric MANOVA (Wilks' Lambda: *F*_24,14_ = 0.013, *p* = 0.003; full MANOVA with SCCs in electronic supplementary material, S3). Randomization MANOVA (9000 randomizations) yielded identical statistical results (*p* = 0.003), suggesting that our conclusions are robust to any departures from distributional assumptions. Based on SCCs, TRFs at low MW (2 kb, SCC = 6.19; 3 kb, SCC = −7.69; and 4 kb, SCC = 7.79) were the largest contributors to the significant multivariate treatment effect (figure 3). SCC absolute values for all other TRFs were low (electronic supplementary material, S3). Multivariate pairwise contrasts indicated that the high-cort treatment group differed from both control (*p* = 0.001) and low-cort groups (*p* = 0.001), but low-cort and control groups did not differ (*p* = 0.57). Taken together, the analyses of TRF distributions show that the high-cort group had an over-representation of short telomeres (less than 5 kb) relative to the low-cort and control groups (figure 3).

## 4. Discussion

We examined the effect of prenatal exposure to elevated yolk cort on the function of the HPA axis and on oxidative stress post-hatch. Embryonic exposure to cort increased the duration of the acute stress response and the proportion of short telomeres in the high-cort group, while baseline ROMs levels were increased in both cort treatment groups. In addition, ROMs increased and TAC decreased over the course of an acute stress response in all birds, regardless of treatment, suggesting that the rise in glucocorticoids during a stress response shifts individuals into oxidative stress.

### (a) Hypothesis 1: prenatal cort exposure alters HPA function during an acute stress response post-hatch

Exposure to prenatal glucocorticoids can modify HPA function through alteration of baseline, total or recovery glucocorticoid levels. In our study, baseline cort did not differ among groups, and we detected a significant treatment × time interaction affecting plasma coricosterone concentration between 15 and 40 min. Although none of the pairwise differences between groups was significant following correction for multiple tests, it appeared that compared with controls, high-cort birds maintained elevated cort levels at 40 min and low-cort birds were intermediate to the other groups (figure 1). This suggests that embryonic exposure to cort in our study altered the trajectory for recovery following an acute stress response. Modifications to HPA activity following prenatal stress can alter the expression of glucocorticoid receptors in the hippocampus [7], which can lead to dysregulation of negative feedback control of glucocorticoid secretion and a protracted glucocorticoid decline after stress [31]. This same impairment may occur in other species, and more comparative work is needed to determine the mechanisms by which prenatal cort exposure modifies HPA function.

HPA development can be affected by the timing, duration and magnitude of glucocorticoid exposure and effects can also be sex-dependent. Numerous studies have examined how prenatal stress alters HPA axis reactivity, providing mixed results (reviewed in [7]). For example, in guinea pigs, a single exposure to a 48 h period of maternal stress resulted in male offspring with reduced baseline and stress-induced HPA activity, but females from the same litter exhibited elevated baseline and stress-induced HPA activity [48]. In another guinea pig study, adult male offspring born to mothers exposed to stress on days 50–52 of gestation exhibited elevated baseline glucocorticoids, while those born to mothers exposed to stress on gestational days 60–62 exhibited normal baseline levels, but heightened stress-induced HPA activity [49]. These studies highlight the complex nature of HPA programming by maternal glucocorticoids. While other mammalian studies also show diverse effects of prenatal stress on HPA axis development, the majority find heightened responsiveness of the HPA axis, including a higher peak and extended duration of glucocorticoid elevation during a stress response in rats [5] and pigs [50], an extended duration of cortisol elevation during a stress response in cows [51], and increased baseline cortisol in monkeys [52] and humans [53].

There have been relatively few studies exploring the effects of prenatal glucocorticoid exposure on the development of the HPA axis in birds. Hayward & Wingfield [13] found that female Japanese quail with cort implants produced offspring with higher HPA axis activity during a stress response. Conversely, later studies in quail [10] and starlings [12] showed that elevating yolk cort by injections resulted in decreased HPA responsiveness of offspring. This contradiction may be explained by differences in the distribution of cort in the egg when injected as opposed to when deposited by the mother [10]. However, since our study also elevated yolk cort through injections, the contradiction suggests other factors may be at play. One difference between our study and the previous yolk injection studies is the dose of cort administered. In quail, cort injection raised overall yolk concentration by 2 standard deviations (from 1.11 to 1.70 ng g^−1^ yolk; [10]), and in starlings, injection raised overall yolk concentration by 1.5 standard deviations (from 15.4 to 28.3 ng g^−1^ yolk; [12]). Our treatments raised overall yolk concentration by 3.5 standard deviations (from 2.13 to 10.21 ng g^−1^ yolk), approximately twice as much as the quail and starling studies, but still well within the normal range found in chicken yolk. This increase seems appropriate as one of the five control eggs used to determine baseline yolk cort levels had a concentration of 7 ng g^−1^ yolk; a value intermediate to our low and high doses. Further, it suggests that the doses used in our study reflect physiologically relevant yolk cort concentrations in chickens. Other studies have reported that the dose of glucocorticoids experienced by developing rats can result in either a hypo- or hyper-active HPA axis in later life (reviewed in [7]). Given that our high-cort, but not low-cort dose affected HPA function, it is possible that the differences found between our study and earlier avian studies may reflect a difference in the amount of cort experienced by the developing embryo.

### (b) Hypothesis 2: prenatal cort exposure impacts cellular ageing processes

Exposure to prenatal glucocorticoids may modify cellular ageing processes through an increase in the generation of ROS, disabling antioxidant defences, or accelerating telomere loss [19,32]. We found that all birds, regardless of treatment group, shifted to oxidative stress over the course of the stress response as illustrated by the concomitant increase in ROMs concentration and decrease in TAC (figure 2). However, initial ROMs were higher in juvenile birds that had experienced elevated prenatal cort (figure 2). Given that we administered cort only once, before incubation, it is surprising to see an effect on relatively short-lived ROMs six weeks later. Given ROMs' relatively short half-life, this suggests that a higher level of ROMs was continuously generated in the birds experiencing elevated prenatal cort. In addition to higher baseline ROMs values, we found that birds experiencing the highest prenatal exposure to cort had an over-representation of short telomeres compared with the control and low-cort groups (figure 3). To our knowledge, this is the first study to show that prenatal exposure to glucocorticoids can increase telomere shortening.

Oxidative stress can be defined as the balance between ROS generation and antioxidant defence. If ROS and antioxidants are in homeostatic balance, oxidative stress is low, but if ROS generation exceeds antioxidant defence, oxidative stress is high, resulting in oxidative damage [17]. For this reason, recent reviews of oxidative stress emphasize the importance of measuring both pro- and antioxidants to avoid making erroneous assumptions about oxidative stress balance [17,54,55]. ROMs are early peroxidation products that form from free radicals and their reactive nature allows them to propagate further oxidative damage. Given ROMs' relatively short half-life, the assessment of both pro-oxidant production via ROMs and antioxidant production via TAC provides a useful indicator of short-term changes in oxidative stress [17,53,54].

All birds, regardless of treatment, shifted to relatively higher levels of oxidative stress response over the course of an acute stress response. The concomitant increase in ROMs concentration and decrease in TAC suggests that the antioxidant system of the chicks could not effectively cope with a sudden overproduction of ROS, as shown in other studies [44]. The acute shift into oxidative stress in response to cort has been shown in mammalian [37,38] and avian studies [33–36], but to our knowledge previous studies have not shown how oxidative stress changes during an acute stress response. Recent *in vitro* work has shown that blocking glucocorticoid receptors via the antagonist RU486 also blocks the glucocorticoid-mediated rise in ROS production, suggesting that glucocorticoids directly regulate genes involved in free-radical generation [56]. Taken together, these results suggest that glucocorticoids may act as modulators of the balance between pro- and antioxidants. More data are needed to determine whether heightened stress responses, longer stress responses or increased frequency of stress responses result in a larger or longer shifts to oxidative stress.

The cause of the elevated baseline ROMs is unclear, but does not appear to be related to higher baseline levels of cort. We do not know how long plasma cort remained elevated after the stress response in our cort-injected birds, and in general, we do not know how long birds remain shifted to oxidative stress after the completion of a stress response. However, if there is a delay in recovery from an acute stressor, this might maintain ROMs at a higher baseline level. Indeed, subjecting chickens to a single injection of cort results in elevated lipid peroxidation in skeletal muscle 48 h afterwards [34]. It is likely that all birds in our study were subjected to daily stress responses caused by handling; as other studies measuring stress responses in young birds in captivity indicate that they do not habituate to frequent handling [57,58]. Therefore, it is plausible that that the higher baseline ROMs in the birds experiencing elevated prenatal cort is due to delayed recovery following frequent acute stress responses.

The pioneering work of Epel *et al*. [15] established that chronic stress in humans resulted in an increased rate of telomere shortening. Further work showed that elevated glucocorticoids were related to negative effects on telomeres, suggesting that stress hormones mediate the destructive effect of stress on telomere maintenance [16]. In our study, the effect of prenatal glucocorticoid exposure on telomeres might be mediated by at least two non-mutually exclusive mechanisms. First, compared with other regions of DNA, telomeres are particularly vulnerable to oxidative damage [19,20]. This may make telomere length, and specifically longitudinal changes in telomere length over the course of months or years, a good indicator of the long-term history of oxidative stress experienced by an individual; although knowledge of genetic determinants and telomere restoration mechanisms should also be taken into account [19,20]. We found that birds with high prenatal exposure to cort had higher levels of ROMs in plasma, suggesting that these birds were constantly experiencing higher levels of oxidative stress that may hasten telomere loss. A second possibility is that glucocorticoids might impact telomere loss through their effects on the telomere-restorative enzyme, telomerase. During acute stress in humans, telomerase levels rise and possibly improve resistance to oxidative stress [59]. However, chronic exposure to cortisol *in vitro* downregulates telomerase activity up to 3 days later by causing a reduction in the transcription of telomerase reverse transcriptase, the catalytic component of telomerase [60]. This is supported by *in vivo* work showing that chronic stress in humans is associated with reduced baseline telomerase activity [15,59]. Further research in this area should use longitudinal telomere measurements to chart the rate and the magnitude of telomere loss and determine the impact of maternal glucocorticoids on telomerase. Given that short telomeres or rapid telomere loss rates are associated with lower survival prospects in humans [21–23] and birds [25–28], our results suggest that maternal glucocorticoids may decrease survival during adult life.

### (c) Conclusion: an evolutionary perspective

Maternal effects can have important and lasting influences on offspring phenotype [61]. Whether maternal effects are adaptive because they convey information on environmental conditions to offspring, or are non-adaptive physiological constraints that can decrease offspring performance has been debated [8], but a new emphasis on how maternal effects might impact both maternal and offspring fitness is emerging [6,62,63]. For example, during poor environmental conditions, yolks from stressed European starling mothers have more cort, which may cause male-biased mortality that ultimately results in maternal fitness gains through increased survival and future fecundity [6].

Maternal glucocorticoids appear to have diverse effects on the development of offspring's HPA function (reviewed above). In birds, maternal glucocorticoids can dampen [10,12] or heighten ([13], this study) HPA function during a stress response. Chicks raised under conditions of unpredictable food delivery suppress baseline and maximum acute stress-induced cort levels [64]. If lower glucocorticoids function to conserve glucose levels when the environment is unpredictable, then one possibility is that dampened HPA activity in response to yolk cort may induce chick physiology to track environmental quality and thus increase chick survival [12,65]. Alternatively, heightened HPA activity can enhance fear and vigilance behaviours in offspring expecting a hostile environment, allowing them to avoid potential predators and stay close to conspecifics [13,65,66], thereby increasing fitness. Sorting out the alternative hypotheses of maternal glucocorticoids on HPA axis sensitivity will begin with a better understanding of the mechanisms by which elevated yolk cort can cause context-dependent heightening or dampening of offspring HPA axis function from a life-history perspective.

Our study showed, for the first time, to our knowledge, that embryonic glucocorticoid exposure increased oxidative stress and accelerated telomere loss. Higher levels of oxidative stress and accelerated telomere loss are both linked to age-related risk factors for disease and increased risk of mortality (reviewed in Monaghan *et al*. [17] and Haussmann & Marchetto [19]). Therefore, one possibility is that the potential benefits to offspring that arise through maternal glucocorticoids may be tempered by costs that are not evident until much later in adult life. However, although maternally derived glucocorticoids may ultimately reduce offspring lifespan, they may increase realized reproductive success of offspring or mothers, and thus still be adaptive [6]. Glucocorticoid-induced phenotypes thus may be postulated to be a context-dependent strategy for maximizing maternal or offspring fitness under changing environmental conditions. Moving forward, integrative work across disciplines will be needed as we continue to probe the causal links among glucocorticoids, cellular ageing and survival.
